# Spatial Lipidomics Reveals Anticancer Mechanisms of Bufalin in Combination with Cinobufagin in Tumor-Bearing Mice

**DOI:** 10.3389/fphar.2020.593815

**Published:** 2021-01-12

**Authors:** Jinghui Zhang, Yanjun Hong, Peisi Xie, Yang Chen, Lilong Jiang, Zhiyi Yang, Guodong Cao, Zhongjian Chen, Xuesong Liu, Yong Chen, Yongjiang Wu, Zongwei Cai

**Affiliations:** ^1^ College of Pharmaceutical Sciences, Zhejiang University, Hangzhou, China; ^2^ Department of Chemistry, State Key Laboratory of Environmental and Biological Analysis, Hong Kong Baptist University, Hong Kong, China; ^3^ Shenzhen Research Institute and Continuing Education, Hong Kong Baptist University, Shenzhen, China; ^4^ School of Pharmaceutical Sciences (Shenzhen), Sun Yat-sen University, Shenzhen, China; ^5^ Cancer Research Institute, Zhejiang Cancer Hospital, Hangzhou, China

**Keywords:** chansu, bufalin, cinobufagin, lipidomics, mass spectrometry imaging

## Abstract

Bufalin (BFL) and cinobufagin (CBF) are the principal bioactive constituents of Chansu, a widely used traditional Chinese medicine (TCM). The synergistic effects of potential active components are responsible for the bioactivities of TCM. Our results showed that the cotreatment with BFL and CBF confers superior anticancer efficacy compared to monotreatment. To reveal the underlying mechanisms of their cotreatment, an integrated method composed of mass spectrometry-based lipidomics and matrix-assisted laser desorption/ionization mass spectrometry imaging was used to delineate the responses of tumor-bearing mice treated with BFL and CBF individually or in combination. The cotreatment with BFL and CBF modulated the sphingolipid metabolism and glycerophospholipid metabolism, and subsequently led to mitochondria-driven apoptosis and systemic disruption of biomembranes in tumor cells. Furthermore, we found that the disturbed lipid markers were mainly located in the non-necrotic tumor areas, the essential parts for the formation of solid tumor framework. Together, our findings revealed what occurred in tumor in response to the treatment of BFL and CBF, from lipids to enzymes, and thus provide insights into the critical role of lipid reprogramming in the satisfactory anticancer effect of BFL in combination with CBF.

## Introduction

Hepatoma is a severe malignancy usually with poor prognosis (Thomas and Zhu, 2005). Although hepatoma can be surgically resected, chemotherapy has an irreplaceable status and function for patients in advanced stages (El-Serag et al., 2008). However, chemotherapy agents are still limited by many disadvantageous factors, such as side effects and drug resistance (Roth et al., 2013; Duncan, 2014). It is urgent to find more effective chemotherapeutic options. Chansu is obtained from the skin secretions of *Bufo bufo gargarizans* Cantor. Its extract has long been used as an anticancer agent in China and other Asian countries (Meng et al., 2009). Huachansu capsules, a sterilized extract of Chansu, has been marketed and used in clinical. They have been widely used for the treatment of patients with various types of cancer, especially in liver cancer (Huang et al., 2020). The synergistic effects of the bioactive components account for the anticancer effects of Chansu and Huachansu. Bufadienolide-type cardiotonic steroids, bufalin (BFL) and cinobufagin (CBF) (Supplementary Figure S1), are the principal bioactive components in Chansu and Huachansu (Qi et al., 2011). We detected BFL and CBF in Huachansu capsules by ultra high performance liquid chromatography (Supplementary Figure S2). Their individual anticancer effects were associated with a downregulation of the pro-survival proteins Bcl-2, and an upregulation of the pro-apoptotic protein Fas and Bax (Qi et al., 2011; Shen et al., 2014). So far, the anticancer mechanisms of BFL and CBF were mostly assessed individually, which were insufficient for the guidance of their clinical joint use. Therefore, more emphasis should be put on the mechanisms of the combination of BFL and CBF.

Lipids play a fundamental role in maintaining membrane homeostasis, providing energy and are involved in cell signaling in all living cells (Ackerman et al., 2018; Storck et al., 2018). Accumulating evidence suggested that cancer was related to aberrant lipid metabolism (Hirsch et al., 2010; Deng and Li, 2020). Rapid proliferating cancer cells required increased lipid biosynthesis for the construction of membrane. And bioactive molecules produced by lipid catabolism acted as signal molecules in the regulation of cancer metastasis (Vander Heiden and DeBerardinis, 2017). As one important subfield of metabolomics, lipidomics investigate the holistic changes of endogenous lipids in response to stimuli based on analytical chemistry principles (Yang and Han, 2016). In view of the important role of lipid metabolism in cancer, lipidomics has been widely used in the diagnosis and treatment of cancer. For the diagnosis of cancer, the disorder of lipid metabolism occurred early in the tumor progression, which made lipids suitable to be used as diagnostic markers (Perrotti et al., 2016). For the treatment of cancer, understanding lipid metabolism pathways in cancer cells could provide potent targets for therapy, and elucidating the function of lipids could benefit the development of new anticancer drugs for clinical evaluation. Determining the lipid change induced by drugs could help to clarify the mechanism of drug action, and could provide a basis for the combination and efficacy improvement of drugs (Zhang et al., 2017; Brovkovych et al., 2018).

In addition to changes in the lipidomics, spatial information is essential to investigate subtle, highly localized changes of metabolites in histopathological regions of tumor. Therefore, advanced imaging techniques are needed for probing focal changes. Mass spectrometry imaging (MSI) is a powerful technique to simultaneously visualize the spatial distribution of molecules in biological samples. MSI has been widely utilized for the diagnostic and prognostic marker discovery (Sun et al., 2019), pharmacological target screening (Luo et al., 2013; He et al., 2015) and the investigation of the spatial distribution of metabolites, lipids and peptides in biological samples (Burnum et al., 2008; Muller et al., 2015; Li et al., 2020). In particular, lipids are appropriate for MSI analysis for several reasons: the polar head groups of many lipid species make their ionization easier; they are abundant components of tissues; and most of them with a molecular weight within 300–1,000 Da (Zemski Berry et al., 2011).

In the present work, an enhanced anticancer effect was demonstrated by the cotreatment with BFL and CBF in the xenograft model. Their synergistic anticancer effect was obtained from cell culture experiments in our unpublished work. Our previous work demonstrated the metabolic regulation effects of the cotreatment of BFL and CBF *in vitro* (Zhang et al., 2020). To thoroughly investigate the underlying molecular mechanisms of their anticancer effects and reveal the possible target region, tumor-bearing mice were treated with BFL and CBF individually or in combination. Thereafter, lipid disturbance analysis was performed by liquid chromatography-mass spectrometry (LC-MS) based lipidomics combined with matrix-assisted laser desorption/ionization mass spectrometry imaging (MALDI-MSI). The findings of this work might provide a new insight to explore the anticancer mechanisms and localize the target region of BFL and CBF on the treatment of hepatoma.

## Method

### Chemicals and Materials

BFL and CBF with a purity over 98% were purchased from Chengdu Must Biotech Co., Ltd. (Chengdu, China). Cisplatin were purchased from Solarbio Biosciences Company (Beijing, China). Sinapic acid (SA), α-cyano-4-hydroxycinnamic acid (HCCA), 2,5-dihydroxybenzoic acid (DHB), peptides and N-(1-naphthyl)-ethylenediamine dihydrochloride (NEDC) were purchased from Sigma-Aldrich (MO). Phosphate-buffered saline (PBS) was purchased from GIBCO (Grand Island, NY). Human hepatoma cell line HepG2 was purchased from Cobioer Biosciences Company (Nanjing, China).

### Establishment of Tumor Nude Mice Model (Tumor Xenografts) and Drug Administration

**FIGURE 1:**
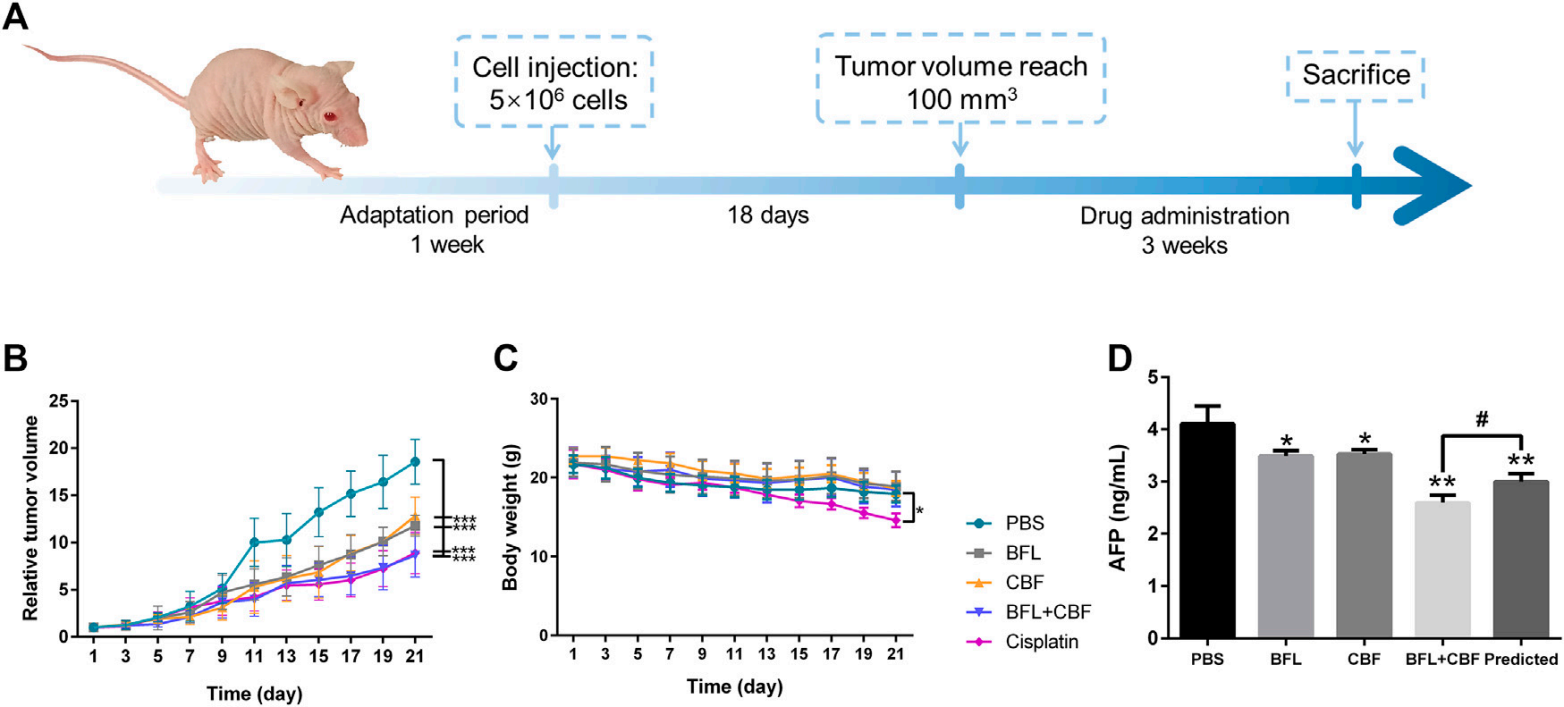
Tumor growth inhibition in HepG2 bearing nude mice after treatment with BFL, CBF and their combination. **(A)** *In vivo* experimental design. **(B)** The growth of relative tumor volume assigned to five experimental groups. **(C)** The change of body weight along with time. **(D)** Serum levels of AFP. All data were represented as mean ± SD. *p < 0.05; **p < 0.01; ***p < 0.001 with PBS group (negative control); #*p* < 0.05; ##*p* < 0.01; ###*p* < 0.001 with cisplation group (positive control) or predicted value.

Male 4–6 weeks-old BALB/c nude mice were purchased from the Chinese University of Hong Kong. All mice were maintained in sterile individually ventilated cages. Water and food were available ad libitum. The lights were on for 12 h per day, and the temperature was kept at 20 ± 2°C with humidity of 45 ± 10%. After 7 days of adaptation period, 5 × 10^6^ of HepG2 cells were subcutaneously implanted into the armpit of each mouse. When the tumor size reached 100 mm^3^, the mice were randomly divided into five groups (eight mice per group), mice in different groups received drugs or PBS by intraperitoneal injection: 1) BFL 2 mg/kg, once a day, 2) CBF 4 mg/kg, once a day, 3) BFL 2 mg/kg + CBF 4 mg/kg, once a day, 4) Cisplatin 3 mg/kg, twice a week (positive control), and 5) PBS (negative control). The body weight and tumor volume were measured every two days. The tumor volume was calculated by the formula: V = 0.5 × a × b^2^, where a and b represent the length and width of the tumor, respectively. After 3 weeks, the mice were sacrificed. Tumors were collected and weighed, then stored at -80°C for LC-MS and MALDI-MSI analysis. The detailed schedule for the xenograft tumor model is shown in Figure 1A.

### Enzyme-Linked Immunosorbent Assay to Measure AFP in Serum

Mouse blood samples were collected and incubated for 30 min at room temperature before centrifuging for 20 min at 2000 g. The supernatant serum was carefully collected after centrifugation. AFP ELISA kit was purchased from Meimian Industrial Co., Ltd. (Jiangsu, China). The serum levels of AFP were measured according to the vendor’s instructions.

### Interaction of Bufalin and Cinobufagin

Bliss independence is widely used to analyze drug interaction. The method compares the observed combination effect (Y_O_) with the predicted combination effect (Y_P_) (Bliss, 1939). Typically, the combination effect is declared synergistic if Y_O_ is greater than Y_P_.

For statistical calculation, the relation between drug effect and concentration of AFP was described as the following equation:

$$\text{Y}=1-\frac{{\text{AFP}}_{\text{treated}}}{{\text{AFP}}_{\text{control}}}$$

where Y is the effect of drugs; AFP_treated_ and AFP_control_ are the concentration of AFP in the serum of treated group and PBS group, respectively.

According to Bliss independence, the combination effect for BFL and CBF can be predicted as:YP=YBFL+YCBF−YBFLYCBFWhere Y_BFL_ and Y_CBF_ are the effect of BFL and CBF, respectively.

### Lipidomics Analysis

Ten mg of tumor tissue was mixed with 320 μL of ice-cold MeOH/H_2_O (80:20, v/v) and homogenized using a Polytron PT2100 homogenizer (Kinematica, Lucerne, Switzerland). Subsequently, 1 ml of MTBE was added and vortexed for 1 min. A total of 200 μL of water was added to induce phase separation. Sample was vortexed for 1 min and incubated at room temperature for 5 min. After centrifugation at 12,000 g and −4°C for 15 min, the upper phase was collected and dried at 4°C.

The lipidomic analysis was performed using an Ultimate 3,000 ultra-high performance liquid chromatograph (UHPLC, Dionex, Sunnyvale, CA) coupled with an Orbitrap Fusion Tribrid mass spectrometer (Thermo Fisher Scientific Inc., Waltham, MA). Lipid separation was performed on an ACQUITY UPLC BEH C18 column (2.1 mm × 100 mm, 1.7 μm, Waters, Milford, MA). The details of mobile phase for UHPLC and MS parameters are summarized in Supplementary Table S1.

The raw data were processed by LipidSearch software (Thermo Fisher Scientifific Inc., Waltham, MA) for extraction, alignment and identification of the lipids. The multivariate statistical analysis was processed using SIMCA software (Version 14.1, Umetrics, Sweden). Orthogonal partial least squares discriminant analysis (OPLS-DA) and Student’s *t*-test were performed. The *p*-value and fold change (FC) were calculated from the peak area. The differential lipids between the control and drug treated groups were selected based on the variable importance in projection (VIP) value (VIP >1), *p* value (*p* < 0.05) as well as fold change (FC > 1.2 or <0.8).

### Quantitative Real-Time Polymerase Chain Reaction

Total RNA was extracted from tumor tissue using the RNAiso plus kit (TaKaRa, Japan). cDNAs were synthesized using the PrimeScript RT reagent kit (Takara, Japan). Quantitative PCR was performed using SYBR Premix Ex Taq (Takara, Japan) on Piko Real-Time PCR system (Thermo Scientific, Waltham, MA). Statistical analysis was conducted by GraphPad Prism five Software, Inc. (La Jolla, CA).

### Lipid Imaging by Matrix-Assisted Laser Desorption/Ionization Mass Spectrometry Imaging

The 14-μm-thick tumor tissue sections were cut using a CryoStar Nx70 cryostat (Thermo Fisher Scientific, Walldorf, Germany) at -20 °C. The slices were dried in a vacuum desiccator for 20 min before MALDI-MSI analysis. Matrix was prepared using an automatic matrix sprayer (ImagePrep, Bruker Daltonics, Billerica, MA), as described by Wang et al. (Wang et al., 2015). Serial tumor tissue sections were subsequently stained using hematoxylin and eosin (H&E) for pathological examination. MALDI-MSI was carried out using a rapifleX MALDI Tissuetyper (Bruker Daltonics, Germany) equipped with a smartbeam laser in the M5 mode. The mass spectra were acquired at a mass range of m/z 250–1,200 in the negative ionization mode by averaging signal from 1,000 shots at 3.0 × 2810 V of detector gain and 82% of laser power. The other parameters were optimized, including lens voltage (11.00 kV), reflector voltage (20.84 kV), pulsed ion extraction time (100 ns) and ion source voltage (20 kV). The spatial resolution for MALDI-MSI was set at 100 μm. The instrument calibration was performed with external standards (SA, DHB, HCCA, and Peptides) before each data acquisition.

The obtained MALDI-MSI raw data were firstly processed and analyzed by FlexImaging 5.0 software (Bruker Daltonics, Germany), and subsequently imported into SCiLS Lab 2016a software (Bruker Daltonics, Germany) for multivariate statistical analysis.

## Results

### Anticancer Effect of Bufalin, Cinobufagin and Their Cotreatment

The antitumor efficacy of BFL, CBF and their combination was investigated in HepG2 tumor-bearing mice using cisplatin as a positive control and PBS as a negative control. As shown in Figure 1B, the tumor volume in the negative control group increased rapidly. When used separately, BFL and CBF displayed a significant tumor growth inhibition. The cotreatment with BFL and CBF led to stronger inhibition of tumor growth, which is similar to the therapeutic efficacy of cisplatin. Weight loss is a serious side effect of chemotherapy that decreases the prognosis of cancer patients (Garcia et al., 2013). Figure 1C showed that neither monotreatment nor cotreatment with BFL and CBF induced significant body weight change for mice. However, we observed the loss of body weight in the cisplatin treated group, which is consistent with previous reports (Garcia et al., 2013; Su et al., 2014). α-fetoprotein (AFP) was one of the most important indicators for hepatoma (Woodfield et al., 2017). Elevated serum levels of AFP have been reported to positively correlate with cancer deterioration (Ortega et al., 2000) Previous study has demonstrated that silencing AFP expression induces apoptosis in hepatoma cell (Yang et al., 2008). As shown in Figure 1D, the predicted concentration of AFP is significantly higher than the observed concentration, that indicating the combination of BFL and CBF induced superior effect than predicted. Their synergetic effect was verified.

### Global Metabolic Shifts Induced by Drug Treatment

**FIGURE 2:**
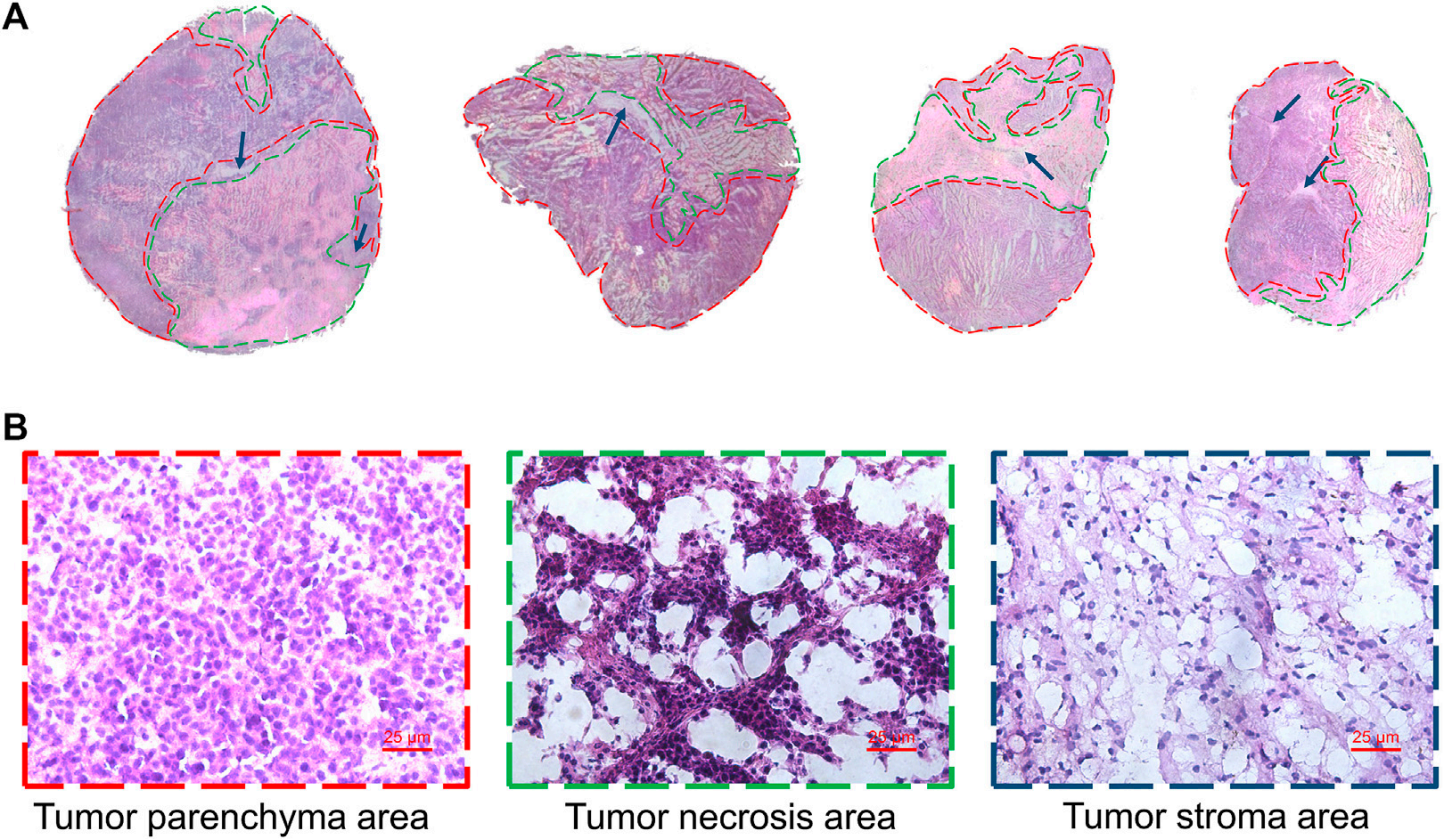
Tumor heterogeneity. **(A)** H&E staining image of tumor tissues. Red frame, green frame and blue arrow represented parenchyma area, necrosis area and stroma areas, respectively. **(B)** The magnification (×40) figure of each representative tumor microregion.

**FIGURE 3:**
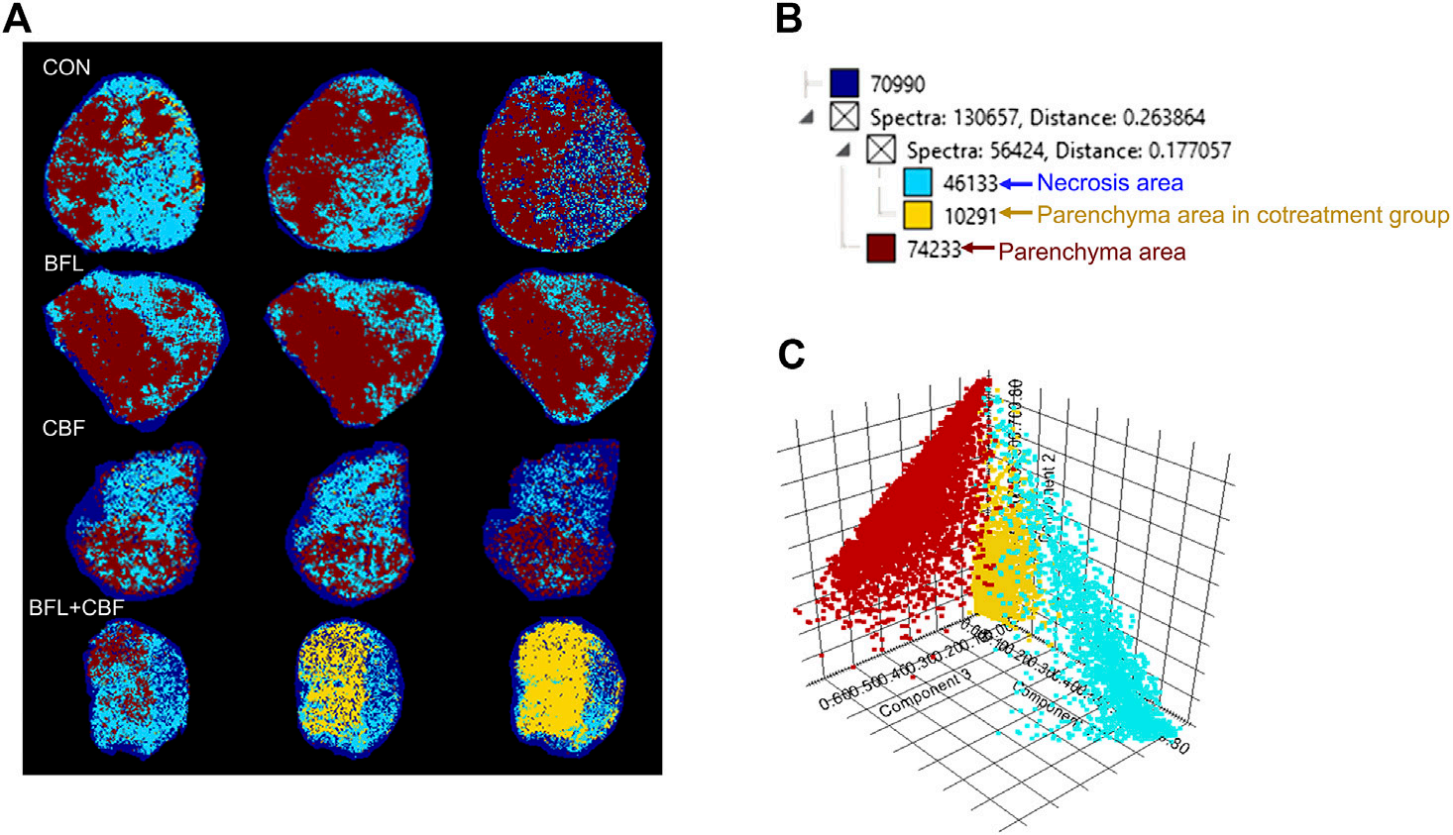
Abundance alteration and spatial distribution of metabolite markers in the tumor from MALDI-MSI analysis. **(A)** Spatial segmentation of the tumor microregion based on MALDI-MSI profiles. **(B)** The dendrogram of segmentation map analysis. **(C)** pLSA score plots for MALDI-MSI profiles.

Heterogeneity is one of the characteristics of malignant tumors, including intratumor morphological diversity and heterogeneity for drug sensitivity (Bae and Park, 2011; Zhang et al., 2014). As shown in Figure 2, the histopathological results indicated considerable spatial heterogeneity in tumor morphology. The parenchyma areas (red frame) and stroma areas (blue arrow) (Bae, 2009), are important in the formation of a firm tumor framework. According to the cell morphology, the tumor necrosis area (green frame) can be determined by the notable cellular debris. To understand the changes in tumor metabolic profile and visualize the histological regions, the spectra from ion signal profiles were processed by SCiLS Lab software and separated using spatial segmentation analysis and probabilistic latent semantic analysis (pLSA). All spectra of the particular cluster were displayed as a spatial segmentation map, and all pixels in the map were colored according to their cluster assignments. As shown in Figure 3A, the bright blue cluster and red cluster corresponded to the tumor necrosis areas and parenchyma areas, respectively. The results of MSI segmentation were consistent with the H&E staining results (Figures 2A,3A). Interestingly, the yellow cluster could only be found in the tumor parenchyma areas of the cotreatment group. According to dendrogram and pLSA (Figures 3B,C), bright blue region and yellow region were separated from the same cluster, indicating their close correlation. The phenomenon might relate to the metabolic dysfunction induced by the cotreatment of BFL and CBF.

### Global Metabolic Shifts Induced by Drug Treatment

**FIGURE 4:**
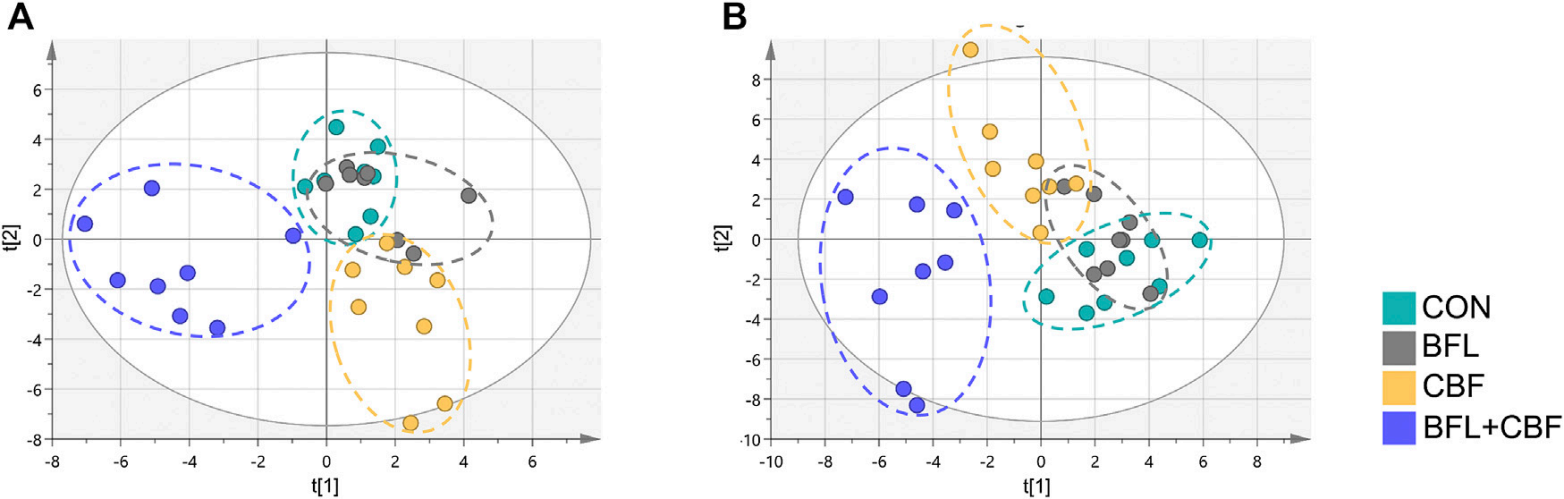
Two-dimensional OPLS-DA score plots of lipidomic profiles. **(A)** Analysis in ESI positive mode. **(B)** Analysis in ESI negative mode.

**FIGURE 5:**
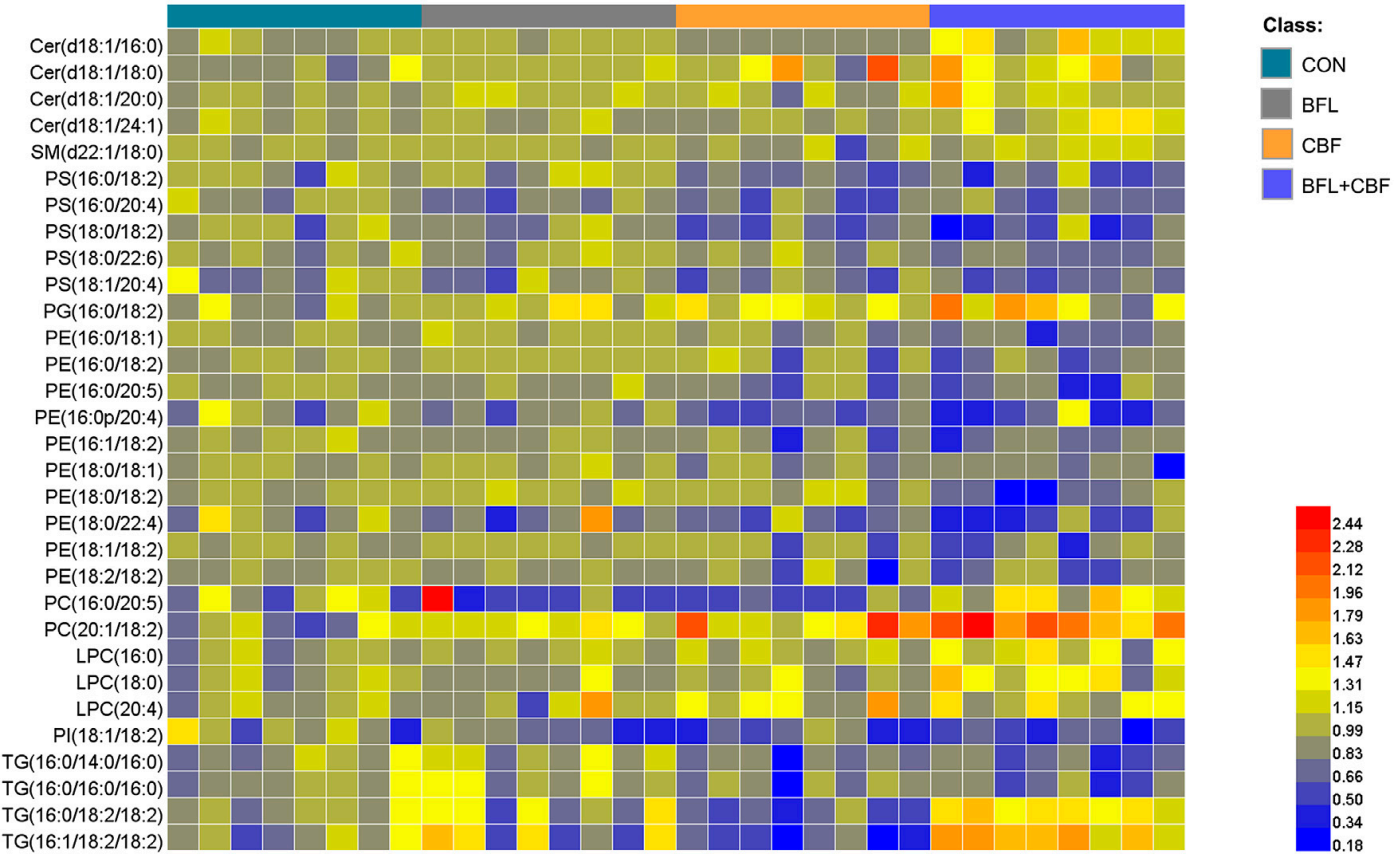
Heatmap analysis of the perturbed lipids follow monotreatment or cotreatment with BFL and CBF. The values were based on the fold change of the lipid peak area (vs. control).

The lipidomic profiles of tumor tissue were acquired using UHPLC-MS/MS under positive and negative ionization modes. 915 ions in ESI (+) and 426 ions in ESI (−) were obtained. OPLS-DA score plots were performed for discriminating between groups. Figure 4 showed the segregation of drug treated groups and the control group. When BFL and CBF were used in combination, distinct differences were observed between cotreatment group and control group, suggesting that the combination of BFL and CBF induced conspicuous perturbation of lipids. A total of 31 perturbed lipids primarily contributed to the separation of BFL + CBF group and control group (Figure 5 and Supplementary Table S2). The main perturbed lipids were sphingolipids (SPs) and glycerophospholipids (GPs).

### Sphingolipid Metabolism

**FIGURE 6:**
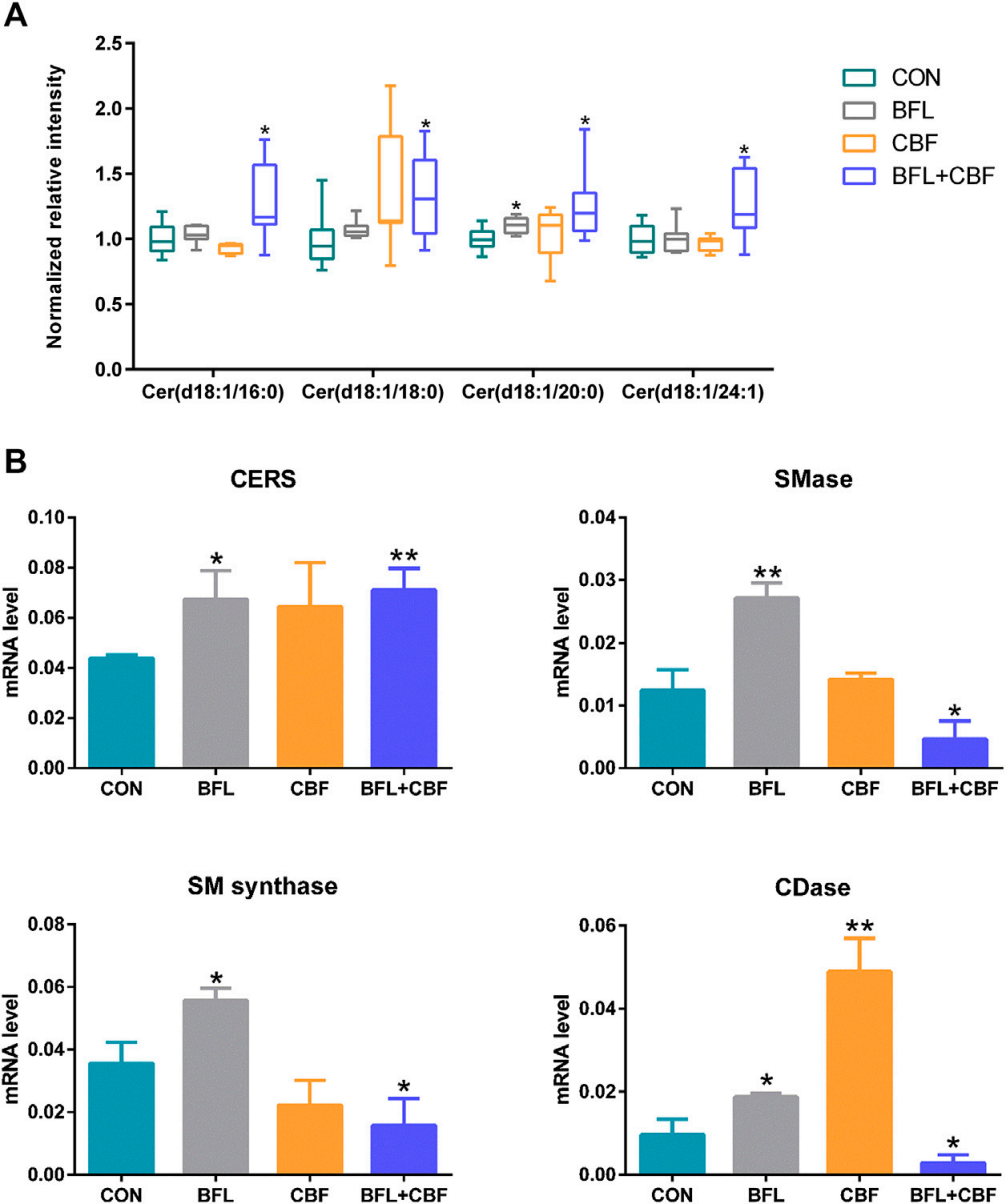
Analysis of SP metabolism following treatment with BFL, CBF and their combination. **(A)** Disturbance of ceramides with different aliphatic chains. **(B)** mRNA expression of genes involved in SP metabolism. Data were represented as mean ± S.D. (*p < 0.05; **p < 0.01 compared to control).

Ceramide (Cer) that played important roles in SP metabolism was significantly increased by BFL + CBF cotreatment (Figure 6A). Cer could be formed either by ceramide synthase (CERS) catalyzed *de novo* synthesis, or through the sphingomyelinase (SMase) dependent hydrolysis of sphingomyelin (SM) (Reynolds et al., 2004). The reaction could proceed in the reverse orientation. The enzyme responsible for SM synthesis was sphingomyelin synthase (SMsynthase), which catalyzed the transfer of a phosphocholine from phosphatidylcholines (PC) to the primary hydroxyl group of Cer forming SM (Ullman and Radin, 1974). The catabolism of Cer proceeded through the action of a ceramidase (CDase), which hydrolyzed the amide bond, thus releasing the sphingoid base and free fatty acid (Mao and Obeid, 2008). As shown in Figure 6B, the cotreatment with BFL and CBF elevated the transcript level of CERS, but suppressed the conversion between Cer and SM. These promoted the biosynthesis of Cer. In parallel to facilitated Cer synthesis, the combination of BFL and CBF suppressed CDase (involved in Cer catabolism). However, the different trend was observed with SMase, SMsynthase and CDase, when BFL and CBF were used individually.

### Glycerophospholipid Metabolism

**FIGURE 7:**
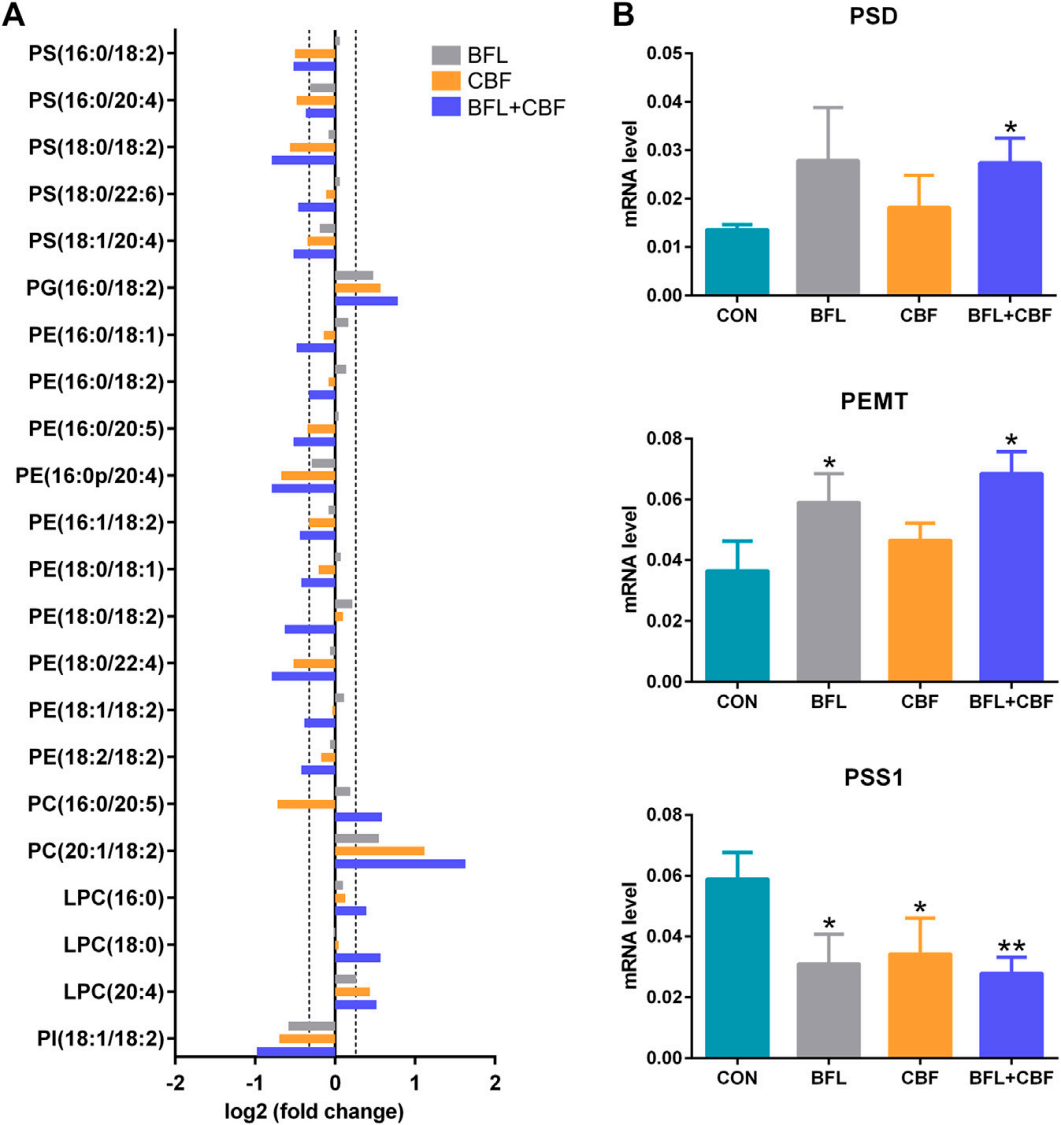
Analysis of GP metabolism following the treatment with BFL, CBF and their combination. **(A)** Fold changes of disturbed GPs (0.8 ≤ FC ≤ 1.2 thresholds were indicated by vertical dotted lines). **(B)** mRNA expression of significantly changed genes involved in GP metabolism. Data were represented as mean ± SD (*p < 0.05; **p < 0.01 compared to control).

Among GPs, the abundance of identified lipids exhibited remarkable upregulation in phosphatidylglycerol (PG), PC and lysophosphatidylcholine (LPC) as well as significant downregulation in phosphatidylserine (PS), phosphatidylethanolamine (PE) and phosphatidylinositol (PI) in the cotreatment group (Figure 7A). To delineate the alteration of key enzymes in the pharmacological actions, the expression of involved genes was determined by qPCR. In GP metabolism, PE was synthesized either by the diacylglycerol ethanolaminephosphotransferase (EPT) catalyzed cytidine diphosphate (CDP)-ethanolamine (Kennedy) pathway or by the phosphatidylserine decarboxylase (PSD) catalyzed PS decarboxylation (Hermansson et al., 2011). All mammalian cells synthesized PC via the 1,2-diacylglycerol cholinephosphosphotransferase (CPT) catalyzed CDP-choline (Kennedy) pathway, but hepatocytes could also produce PC by phosphatidylethanolamine N-methyltransferase (PEMT) catalyzed methylation of PE (Vance et al., 1997). PS was synthesized by phosphatidylserine synthase (PSS) one and two from PC and PE, respectively (Vance and Steenbergen, 2005). Phosphatidylglycerol synthase (PGS) catalyzed the reaction involved in the synthesis of PG (Huang and Freter, 2015). As shown in Figure 7B, the cotreatment of BFL and CBF upregulated the expression of PSD and PEMT, and downregulated the expression of PSS1. However, the expression of EPT, CPT, PSS2 and PGS were not significantly changed (Supplementary Figure S3).

### Visualization and Localization of Anticancer Lipid Biomarkers

**FIGURE 8:**
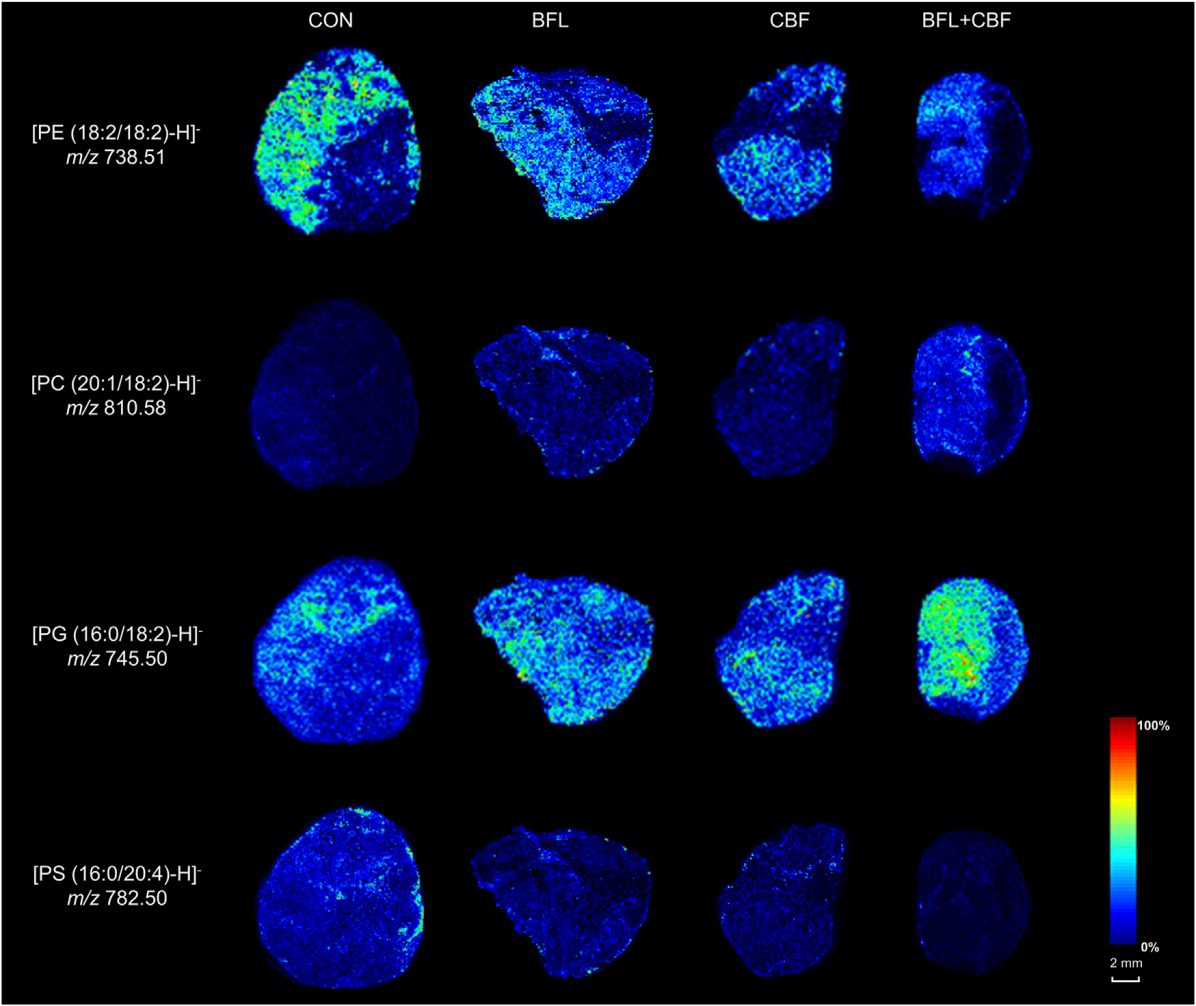
MALDI-MSI images of lipid markers in tumor sections.

MALDI-MSI was used to visualize the spatial distribution of anticancer lipid markers in tumor sections (Figure 8). With the treatment of BFL and CBF individually or in combination, four lipids were found to be significantly altered, which mainly distributed in the parenchyma areas and stroma areas of the tumor. Compared with control group, ion intensities of PC (20:1/18:2) and PG (16:0/18:2) were significantly increased, whereas decreased abundances of PE (18:2/18:2) and PS (16:0/20:4) were found.

## Discussion

Hepatoma is a severe malignancy in the world with high morbidity and mortality. Despite all the medical efforts, it remains radically incurable, especially for advanced cases (Schütte et al., 2014). The low efficiency of single agent is an important reason for this unfavorable situation. Combination therapy has been successfully applied in reducing side effects and achieving enhanced effectiveness (Lehár et al., 2009). The data presented herein showed that the cotreatment with BFL and CBF enhanced the antitumor efficacy in the xenograft mouse model but did not induced significant loss in body weight. As a typical side effect of chemotherapy, weight loss could negatively influence treatment outcomes, even in overweight people (Arends et al., 2017).

With satisfactory efficiency and low side effect, the combination of BFL and CBF was suitable for cancer therapy. To thoroughly clarify their pharmacological mechanisms, a spatial lipidomics approach that aligns well with the complexity and integrity of combination therapy was used to provide insights into the responses of tumor toward the drug combination treatment. The spatial lipid shifts induced by drug treatment were visualized by MALDI-MSI. Interestingly, tumor parenchyma area in the cotreatment group showed similar metabolic profile with necrosis areas of other groups. But the metabolic profile between parenchyma area and necrosis areas are totally different in the monotreatment group (Figures 2, 3). This indicated the transformation of tumor parenchyma areas to necrosis areas, which could benefit the therapy and prognosis of cancer.

**FIGURE 9:**
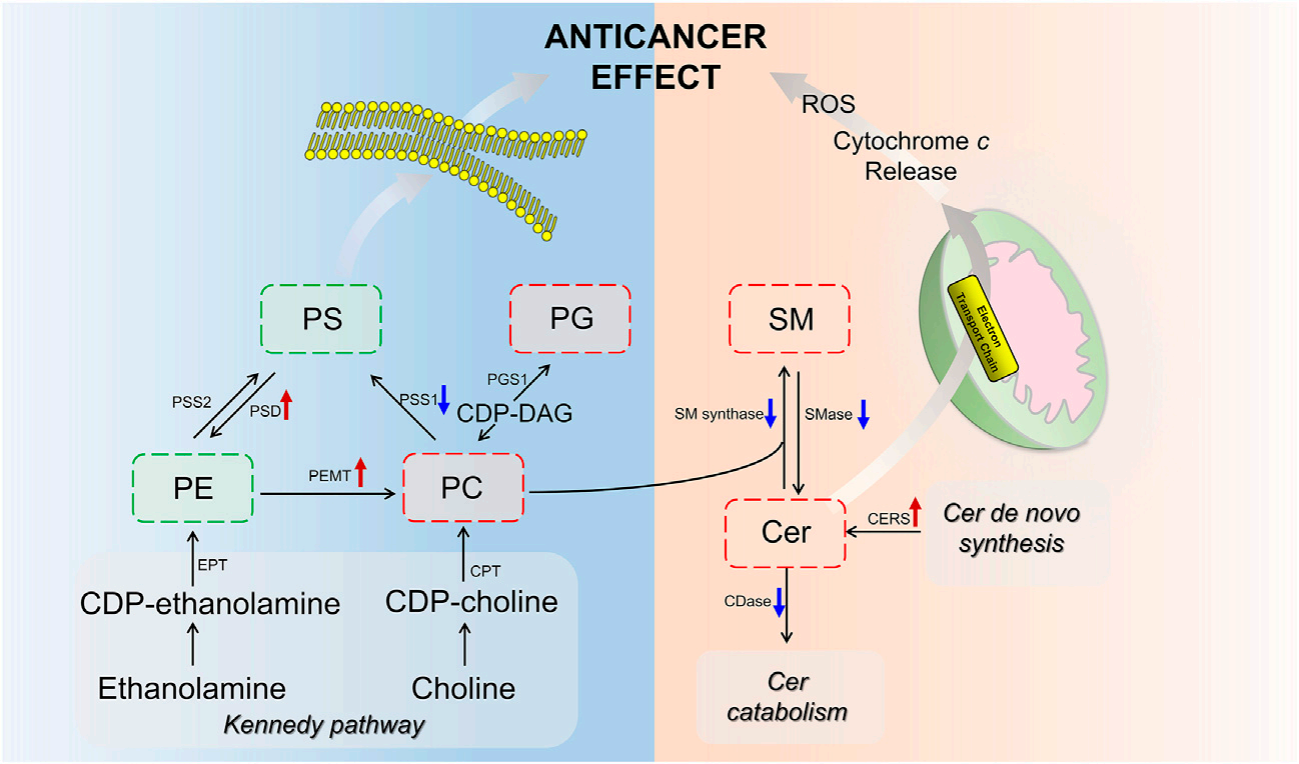
Scheme of perturbed lipid pathways. Lipids and genes marked in red (up arrow) and green (down arrow) represented the up- and downregulation, respectively.

LC-MS based lipidomics was performed to find the lipid markers. The disorder of SPs and GPs was found to have a close relationship with the satisfactory anticancer effect of BFL in combination with CBF:

Of particular interest in lipidomic results was the accumulation of ceramides in SP metabolism (Figure 6A) due to the intimate connection between ceramides and apoptosis (D Mullen and M Obeid, 2012; Li et al., 2014). The stimulated expression of Cer synthesis related gene and the suppressed expression of catabolism related gene jointly led to the accumulation of Cer in the contreatment group. Moreover, sufficient amounts of SM supported Cer biogenesis, although their interconversion catalyzed by SMases and SMsynthase was not upregulated. Increased Cer level was observed in response to many cancer chemotherapeutic agents, including fludarabine, vincristine, etoposide, daunorubicin, irinotecan, paclitaxel, fenretinide and doxorubicin (Senchenkov et al., 2001). Ceramide triggered the mitochondria-driven apoptosis (Won and Singh, 2006). Specifically, ceramide induced the release of cytochrome *c*, an electron carrier of the mitochondrial electron-transport chain (Andrieuabadie et al., 2001; Ow et al., 2008). The release of cytochrome *c* led to a decrease in mitochondrial inner transmembrane potential (ΔΨm), mitochondrial oxygen consumption and Ca^2+^ retention, and all of which caused mitochondrial dysfunction and ROS generation, ultimately induced apoptosis (Ghafourifar et al., 1999) (Figure 9). Our findings indicated that the pro-apoptosis effect of BFL in combination with CBF was mediated in part through the accumulation of ceramide. The inconsistent regulation between monotreatment and cotreatment groups on the conversion between Cer and SM as well as the catabolism of Cer might be a potential mechanism for their synergistic effect.

GPs are the major structural lipids, which elicit crucial biological functions in membrane integrity and functional lipid biosynthesis (Hishikawa et al., 2014). After the cotreatment with BFL and CBF, the abundance of PC was increased, whereas the levels of PE and PS were decreased. This finding indicated that the cotreatment with BFL and CBF promoted the methylation from PE to PC, but not the conversion from PC to PS, which was verified by the activation of PEMT and suppression of PSS1. Besides, PC was also the substrate for SMase to form SM in SP metabolism, and the inhibition of SMase contributed to the accumulation of PC (Figure 9). Consequently, the level of PC was increased. PC induces apoptosis of hepatoma cells via death ligands (Sakakima et al., 2009). Additionally, PE and PC were the major constituents of biological membranes, and contributed to cell proliferation (Gibellini and Smith, 2010). The metabolic disorders of PE and PC might lead to the disruption of cell membrane homeostasis (Vance, 2013). PG was a biosynthetic precursor of cardiolipin, located in the inner mitochondrial membrane and was required for the function of many of the respiratory and ATP synthesizing enzymes (Struzik et al., 2020). The increase of PG suggested that the treatment of BFL and CBF might disturb the structure of mitochondrial membrane. Our results showed that the treatment of BFL and CBF induced a dysregulation of GP metabolism in the tumor, which might result in the instability of biomembranes and produce anticancer efficacy (Figure 9).

To verify the variation of lipid abundance and investigate the therapeutic target of BFL and CBF, we used MALDI-MSI to visualize the spatial distribution of lipid markers in tumor tissue. We found the disturbed lipid markers mainly located in the tumor parenchyma areas and stroma areas. It demonstrated the tumor parenchyma areas and stroma areas (essential parts for the formation of solid tumor framework) were sensitive targets for BFL and CBF (Figures 2, 8). The spatial distribution of drugs in tumor is related to tumor heterogeneity and influencing clinical outcomes (De Maar et al., 2020). The distribution of many drugs was detected by MSI (Lukowski et al., 2017; Strydom et al., 2019). Giordano et al. (Giordano et al., 2016) measured the distribution of paclitaxel, a wildly used anticancer drug, in xenograft mouse model. Mice were treated with paclitaxel intravenously at a single dose of 60 mg/kg. They found paclitaxel accumulated in the non-necrotic tumor areas. However, in our research, mice were treated with 2 mg/kg of BFL and 4 mg/kg of CBF via intraperitoneal injection for 21 days. The doses were too low to be detected in the tumor tissue. In our future work, experiments with higher doses will be performed for the research of the distribution of BFL and CBF in the tumor.

## Conclusion

Together, the present study demonstrated that the combination of BFL and CBF acted synergistically in inducing apoptosis and inhibiting growth in xenograft tumor. A novel mass spectrometry-based spatial lipidomic method was applied to reveal the underlying mechanism. As indicated by MALDI-MSI study, the drugs may penetrate into tumor and act in non-necrotic tumor areas. Our results indicated that the metabolism dysregulation of SPs and GPs with the treatment of BFL and CBF led to mitochondria-driven apoptosis and systemic disruption of biomembranes. In particular, the discrepant regulation of related enzymes in sphingolipid metabolism among the monotreatment and cotreatment with BFL and CBF might account for their synergism. This study provides theoretical basis for the combination of BFL and CBF in clinical practice.

## Data Availability Statement

The raw data supporting the conclusion of this article will be made available by the authors, without undue reservation, to any qualified researcher.

## Ethics Statement

The animal study was reviewed and approved by Hong Kong Special Administrative Region Department of Health (License number: (19-32) in DS/SHS/8/2/6 Pt.3).

## Author Contributions

YW and ZWC conceived the idea. YH designed the study. JZ performed the experimental work and drafted the manuscript. PX conducted the MSI analysis. LJ participated in the animal experiment. YaC and ZJC participated in data analysis. ZY and GC participated in the lipidomic analysis. XL and YoC revised the manuscript. All authors read and approved the final manuscript.

## Funding

This work was supported by the National Major Scientific and Technological Special Project for “Significant New Drugs Development” (2018ZX09201010) and the National Natural Science Foundation of China (21806136).

## Conflict of Interest

The authors declare that the research was conducted in the absence of any commercial or financial relationships that could be construed as a potential conflict of interest.
