# Supplementary material for: Spatial Lipidomics Reveals Anticancer Mechanisms of Bufalin in Combination with Cinobufagin in Tumor-Bearing Mice
Source: Front Pharmacol. 2021 Jan 12;11:593815. doi: 10.3389/fphar.2020.593815 (PMC7883642; doi:10.3389/fphar.2020.593815)
Supplement: Supplementary file 1 [file datasheet1.docx]

Supplementary Material

***Contents Page***

**Supplementary Methods**

Determination of the content of BFL and CBF in Huachansu 2

**Supplementary Figures**

**Supplementary Figure 1** The chemical structures of bufalin and cinobufagin 3

**Supplementary Figure 2** HPLC chromatogram of bufalin and cinobufagin 4

**Supplementary Figure 3** Expression of GP metabolism related genes following the treatment

with BFL, CBF and their combination 5

**Supplementary Tables**

**Supplementary Table 1** Instrumental method for the lipidomic analysis by UHPLC-Orbitrap

Fusion MS 6

**Supplementary Table 2** Significantly dysregulated lipids in tumor tissue 7

**Determination of the content of BFL and CBF in Huachansu**

Accurately weighed (about 2.5 g) Huachansu capsule content was mixed with 20 mL of methanol and weighed. The sample was extracted under a reflux condenser for 1 h. Methanol was added to fit the original weight when samples cooled down to room temperature. The filtered extract of Huachansu capsule content was used for analysis. Analysis was carried out according to the Chinese Pharmacopoeia. Briefly, an aliquot (10 µL) of the filtered sample was injected into Agilent 1290 Series UHPLC system (Agilent Technologies, USA). Chromatographic separation was performed on a Waters Xbridge™ shield RP18 column (250 mm × 4.6 mm, 5 μm). The mobile phase was composed of 0.5% potassium dihydrogen phosphate aqueous solution (pH 3.2) and acetonitrile (50:50, v/v) with isocratic elution. The flow rate was set at 1 mL/min and the detection wavelength was set at 296 nm. The column temperature was kept at 40 ℃.


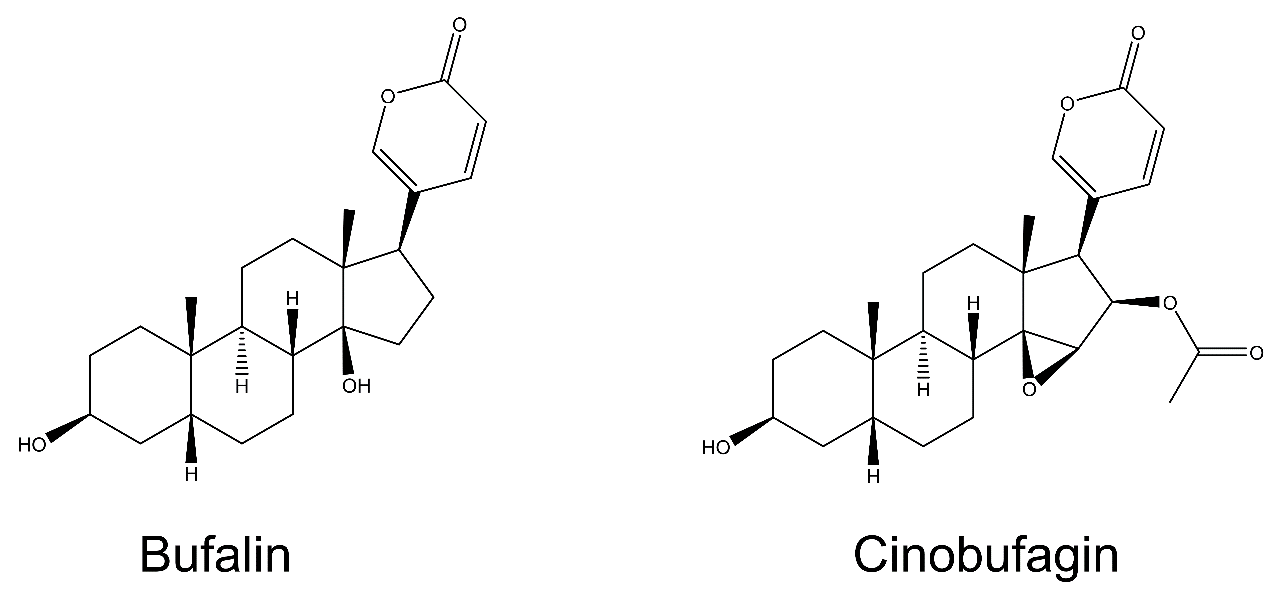


**Supplementary Figure 1.** The chemical structures of bufalin and cinobufagin.


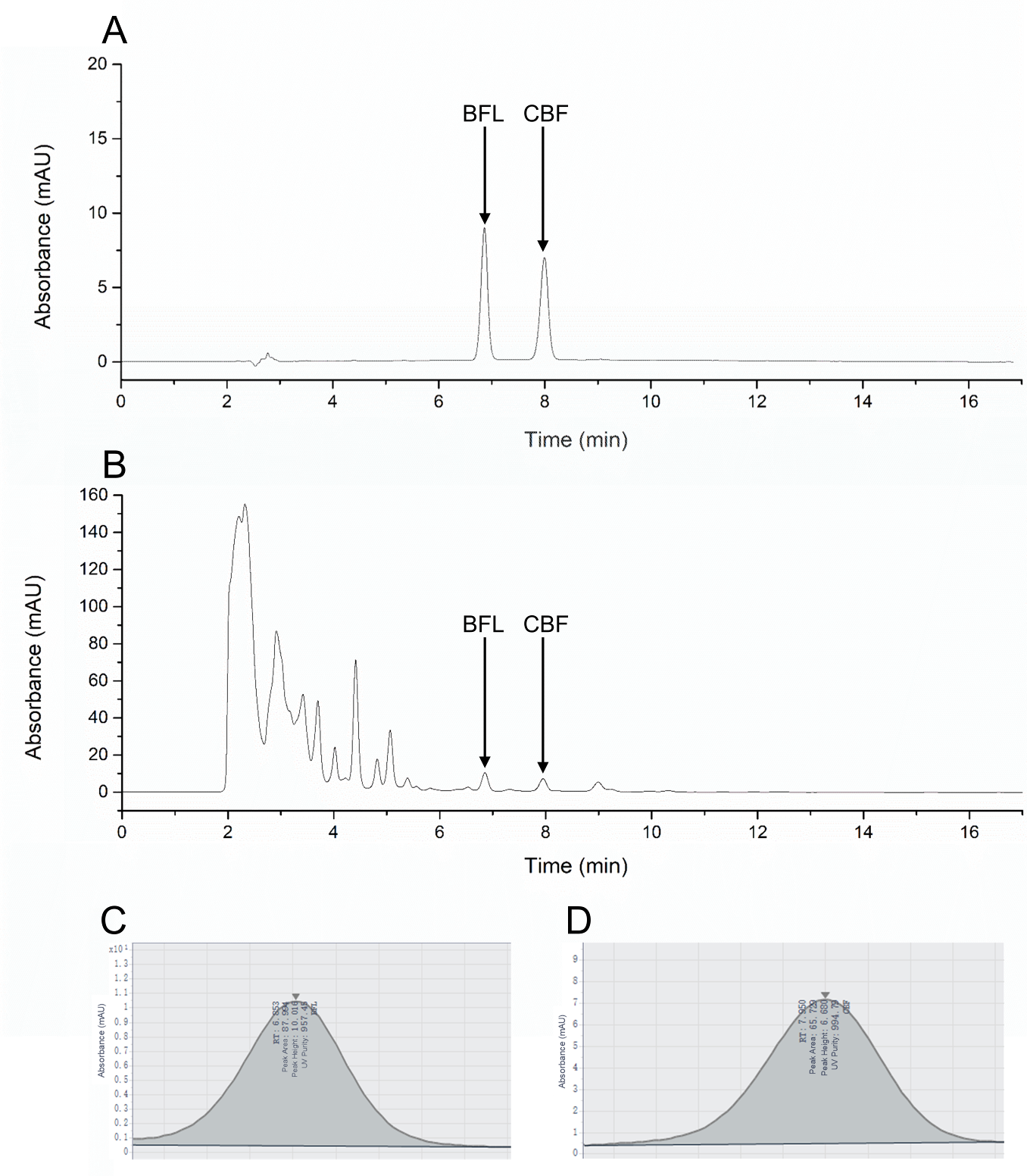


**Supplementary Figure 2.** HPLC chromatogram of bufalin and cinobufagin. (A) chromatogram of standard substances. (B) chromatogram of Huachansu. (C) peak purity of bufalin in Huachansu. (D) peak purity of cinobufagin in Huachansu.


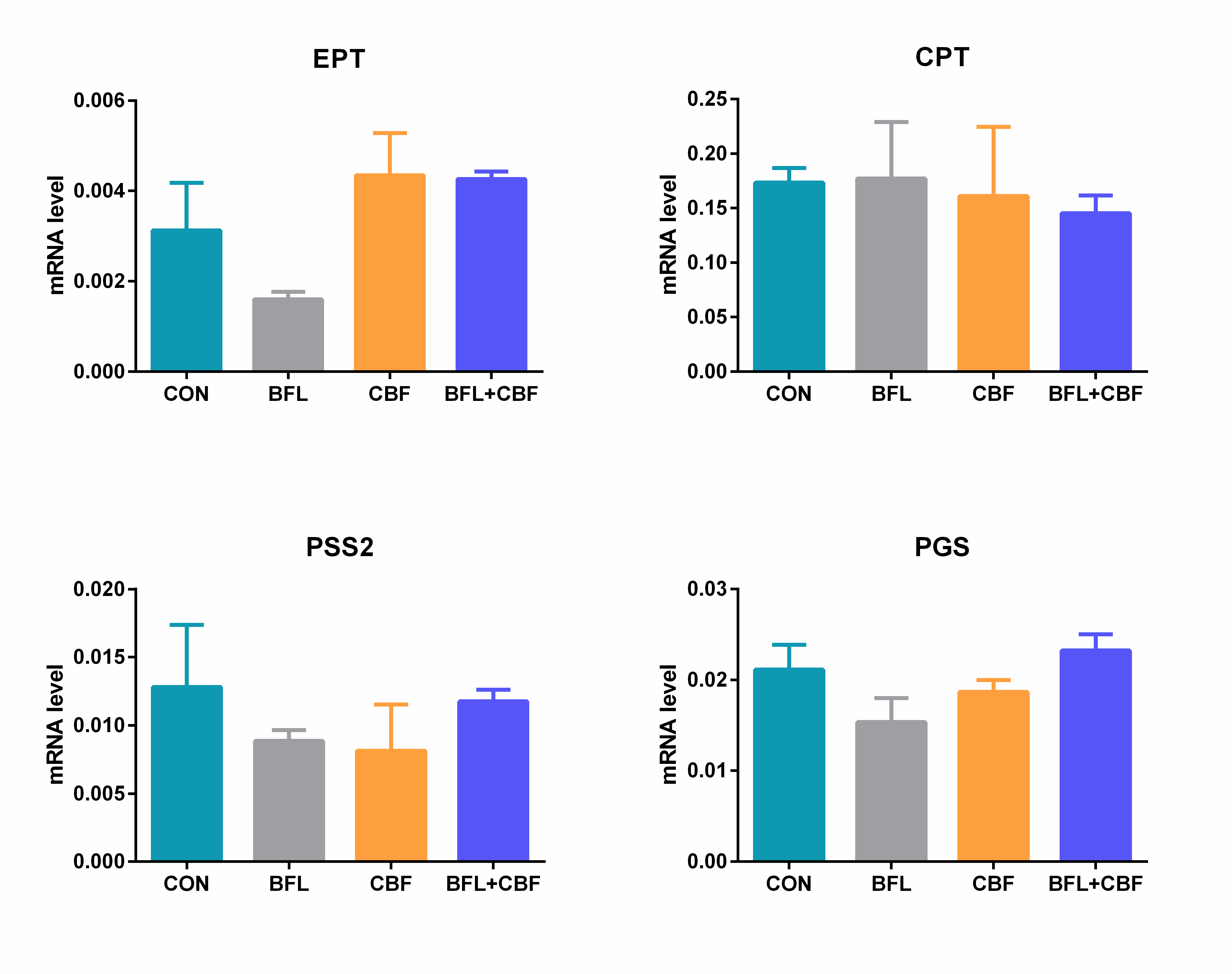


**Supplementary Figure 3.** Expression of GP metabolism related genes following the treatment with BFL, CBF and their combination.

**Supplementary Table 1.** Instrumental method for the lipidomic analysis by UHPLC–Orbitrap Fusion MS.

| Mobile Phases | A: 10 mM ammonium formate and 0.1% formic acid in 2-propanol/acetonitrile (90:10, v/v) | | |
| --- | --- | --- | --- |
|  | B: 10 mM ammonium formate and 0.1% formic acid in acetonitrile/water(60:40, v/v) | | |
| Gradient Profile | Time (min) | Percentage A (%) | Flow Rate (mL/min) |
|  | 0 | 30 | 0.26 |
|  | 1 | 30 | 0.26 |
|  | 2 | 45 | 0.26 |
|  | 7 | 70 | 0.26 |
|  | 9 | 85 | 0.26 |
|  | 17 | 100 | 0.26 |
|  | 19 | 100 | 0.26 |
|  | 20 | 30 | 0.26 |
|  | 23 | 30 | 0.26 |
| Injection Volume | 10 μL | | |
| MS Parameters | Spray voltage (kV) = 3.0 (for both positive and negative ionization mode); | | |
|  | Sheath gas (arbitrary units) = 50; | | |
|  | Auxiliary gas (arbitrary units) = 15; | | |
|  | Ion transfer tube temperature (°C) = 285; | | |
|  | Vaporizer temperature (°C) = 300. | | |
|  | Orbitrap resolution = 30000. | | |

**Supplementary Table 2.** Significantly dysregulated lipids in tumor tissue.

| Lipid | Class | BFL+CBF vs. CON | | | FC (BFL  vs. CON) | FC (CBF  vs. CON) |
| --- | --- | --- | --- | --- | --- | --- |
|  |  | *P* value | VIP | FC |  |  |
| Cer(d18:1/16:0) | Cer | 2.93E-02 | 1.33 | 1.47 | 1.05 | 0.91 |
| Cer(d18:1/18:0) | Cer | 3.66E-02 | 1.34 | 1.54 | 1.11 | 1.68 |
| Cer(d18:1/20:0) | Cer | 3.58E-02 | 1.37 | 1.43 | 1.15 | 1.07 |
| Cer(d18:1/24:1) | Cer | 2.48E-02 | 1.46 | 1.42 | 1.01 | 0.96 |
| SM(d22:1/18:0) | SM | 2.16E-02 | 1.52 | 1.21 | 1.08 | 0.97 |
| PS(16:0/18:2) | PS | 4.37E-02 | 1.33 | 0.70 | 1.04 | 0.71 |
| PS(16:0/20:4) | PS | 3.30E-02 | 1.45 | 0.78 | 0.81 | 0.72 |
| PS(18:0/18:2) | PS | 1.97E-02 | 1.49 | 0.58 | 0.95 | 0.68 |
| PS(18:0/22:6) | PS | 3.97E-03 | 1.70 | 0.73 | 1.04 | 0.93 |
| PS(18:1/20:4) | PS | 4.06E-02 | 1.28 | 0.70 | 0.88 | 0.79 |
| PG(16:0/18:2) | PG | 3.22E-02 | 1.33 | 1.72 | 1.39 | 1.48 |
| PE(16:0/18:1) | PE | 5.90E-03 | 1.63 | 0.72 | 1.12 | 0.91 |
| PE(16:0/18:2) | PE | 3.59E-02 | 1.35 | 0.80 | 1.10 | 0.95 |
| PE(16:0/20:5) | PE | 1.11E-02 | 1.49 | 0.70 | 1.03 | 0.79 |
| PE(16:0p/20:4) | PE | 4.25E-02 | 1.47 | 0.58 | 0.82 | 0.63 |
| PE(16:1/18:2) | PE | 4.63E-03 | 1.68 | 0.74 | 0.95 | 0.80 |
| PE(18:0/18:1) | PE | 2.31E-02 | 1.53 | 0.75 | 1.05 | 0.87 |
| PE(18:0/18:2) | PE | 3.64E-03 | 1.67 | 0.65 | 1.16 | 1.07 |
| PE(18:0/22:4) | PE | 4.11E-02 | 1.39 | 0.58 | 0.96 | 0.70 |
| PE(18:1/18:2) | PE | 2.95E-02 | 1.41 | 0.77 | 1.08 | 0.98 |
| PE(18:2/18:2) | PE | 3.71E-02 | 1.32 | 0.75 | 0.96 | 0.89 |
| PC(16:0/20:5) | PC | 3.72E-02 | 1.43 | 1.50 | 1.14 | 0.61 |
| PC(20:1/18:2) | PC | 2.21E-05 | 2.14 | 3.09 | 1.46 | 2.17 |
| LPC(16:0) | LPC | 4.87E-02 | 1.24 | 1.31 | 1.07 | 1.09 |
| LPC(18:0) | LPC | 1.76E-02 | 1.48 | 1.48 | 0.99 | 1.03 |
| LPC(20:4) | LPC | 2.83E-02 | 1.37 | 1.43 | 1.20 | 1.35 |
| PI(18:1/18:2) | PI | 2.05E-02 | 1.44 | 0.51 | 0.67 | 0.62 |
| TG(16:0/14:0/16:0) | TG | 4.16E-02 | 1.48 | 0.66 | 1.11 | 0.76 |
| TG(16:0/16:0/16:0) | TG | 4.66E-02 | 1.47 | 0.69 | 1.20 | 0.80 |
| TG(16:0/18:2/18:2) | TG | 4.38E-02 | 1.27 | 1.76 | 1.23 | 0.62 |
| TG(16:1/18:2/18:2) | TG | 4.48E-02 | 1.29 | 2.17 | 1.30 | 0.50 |
